# Evaluation of the transcutaneous posterior tibial nerve stimulation in the treatment of life long premature ejaculation: A randomized controlled trial

**DOI:** 10.1080/20905998.2025.2500896

**Published:** 2025-05-11

**Authors:** Mahmoud F. Ghaly, Abdelrahman Ahmed Aburahma, Omar Abdulsalam Azzazi, Hamed Abdalla Hamed, Amr Moubasher, Aliaa Ali Tawfeek, Hesham Nafia, Ahmed Mourad, Ahmed Gamal

**Affiliations:** a Department of Andrology, Sexology & STIs, Faculty of Medicine, Cairo University, Cairo, Egypt; b Department of Dermatology and Andrology, Assiut University, Assiut, Egypt; c Department of Urology, Cardiff and Vale University Hospital, Cardiff, Wales, UK; d Department of Clinical Neurophysiology, Faculty of Medicine, Cairo University, Cairo, Egypt; e Department of Dermatology, Faculty of Medicine, Cairo University, Cairo, Egypt

**Keywords:** Premature ejaculation, IELT, TPTNS, TENS

## Abstract

**Introduction:**

Premature ejaculation (PE) is considered one of the most prevalent sexual dysfunctions among men. The currently approved treatments sometimes have intolerable side effects.

**Aim:**

We conducted a single blinded randomized controlled trial to assess the efficacy and safety of transcutaneous posterior tibial nerve stimulation (TPTNS) for the treatment of lifelong PE.

**Methods:**

Between 2022 and 2023, 50 male patients complaining of lifelong PE were randomized into two equal blinded groups. One group underwent TPTNS while the second group underwent sham transcutaneous electrical nerve stimulation (TENS). TPTNS group underwent 10 sessions of electrical stimulation of the posterior tibial nerve three times per week for 3 weeks using cup electrodes just behind the medial malleoli, while sham TENS group underwent same sessions schedule but with electrodes behind lateral malleoli with underpowered parameters. The Arabic index of premature ejaculation and the intra-vaginal ejaculatory latency time (IELT) measured by the patients’ digital hand watch were our primary outcome. Both were recorded before starting sessions (baseline), at the end of the study (after 3 weeks) and lastly after 2 months of discontinuation.

**Results:**

Sixteen patients experienced improvement in their AIPE score in TPTNS group (64%) compared to 4 in the sham TENS group (16%) (*p* = 0.001). Also IELT significantly improved after receiving TPTNS with median IELT fold increase of 3.1 compared to 1.2 fold increase in sham TENS group (*p* = 0.01).

**Conclusion:**

TPTNS is a potentially promising procedure to treat PE being safe, non-invasive, and clinically effective.

## Introduction

Lifelong premature ejaculation (PE) is defined according to the second international society for sexual medicine (ISSM) Ad Hoc Committee as a triad of : (1) Ejaculation that always or mostly occurs before or within 1 min of penetration of the vagina from the first sexual act; (2) The lack of ability to delay ejaculation on all or most of vaginal penetrations; and (3) Bothersome personal consequences, such as distress, frustration, and/or the evasion of sexual intimacy [1]. The same committee suggested that the 1-min intra-vaginal ejaculatory latency time (IELT) cutoff point should not be rigidly applied as about 10% of patients seeking treatment for lifelong PE have IELT of 1–2 min [1–5].

Several therapeutic modalities for this condition have been tried, such as behavioral therapy, psychotherapy, and pharmacological treatment including local anesthetics, tricyclic anti-depressants, selective serotonin reuptake inhibitors (SSRIs), and phosphodiesterase type 5 inhibitors (PDE-5i) [6–10]. The currently recommended treatment is dapoxetine which has several drawbacks such as lack of spontaneity, cessation of clinical effect after discontinuation, as well as some bothersome side effects including tiredness, insomnia, diarrhea, nausea, headache, and dizziness [6–9]. These drawbacks leave the door open for development of new therapeutic modalities.

Ejaculation consists of two phases, emission and expulsion, and the process depends on the synchrony of both sympathetic and parasympathetic systems [11–14].

The emission phase is mediated by the sympathetic system (T12-L1) that is characterized by closure of the bladder neck sphincter, followed by the accumulation of secretions in the posterior urethra. The expulsion phase is controlled by the sacral parasympathetic and somatic centers (S2–S4) in which the semen is propelled through the rhythmic contractions of the pelvic, bulbospongiosus, and ischiocavernosus muscles [13,14].

The posterior tibial nerve is a large diameter mixed (sensory and motor) nerve containing fibers with medullary origins in the sacral plexus, as does the innervation of the structures of the pelvic floor [15–17].

Transcutaneous posterior tibial nerve stimulation (TPTNS) therapy has extensive application in pelvic floor physiotherapy [18]. It has also been used in treating overactive bladder, fecal incontinence, and primary dysmenorrhea for years [19].

The underlying principle of electrostimulation therapy is based on the complex sensorimotor function of the posterior tibial nerve, originating from T4–S3 roots. While the emission phase of ejaculation is primarily governed by stimuli from the T12–L1 area [20], the expulsion phase is predominantly regulated at the S2–S4 level [11,21]. Consequently, TPTNS has the potential to inhibit both the emission (through the sympathetic system) and expulsion (through the parasympathetic–somatic ejaculation system) phases of ejaculation [22].

## Methods

Asingle-blinded interventional randomized controlled trial (RCT) was conducted with an allocation ratio of 1:1 in the outpatient clinic of Andrology Department, Kasr Al Ainy Hospital, Cairo University in the period between 2022 and 2023 to assess the safety and efficacy of TPTNS therapy to control the ejaculatory reflex for cases with lifelong PE. This study was approved by the local ethical committee of Cairo University with registration code: md-95–2022. This study was registered with ClinicalTrials.gov (NCT06570512).

The participants in this study were recruited from adult males attending the andrology clinic complaining of lifelong PE with ejaculatory latency less than 2 min (85% of them were less than 1 min). They needed to be in a stable and continuous marital relationship, with a frequency of intercourse at least once per week. They were not on any treatment for PE during the period of 21 days before starting the study.

Patients were excluded if they had erectile dysfunction [measured by the International Index of Erectile Function (IIEF-5) questionnaire] [23,24], on current medications for PE, decreased libido, hypogonadism, suppressed male orgasm, alcohol or drug use, inserted pacemaker or heart defibrillator, associated epilepsy or seizures, signs of venous insufficiency or cutaneous wounds or lesions on lower limbs, congenital or acquired anatomical anomalies of the penis diagnosis of ongoing genitourinary tract infection, diagnosis of mental disorders affecting ejaculatory function (depression, anxiety, schizophrenia … etc.), or diagnosis of uncontrolled physical illnesses (hepatic, renal, cardiac, neurological, endocrinal … etc.).

Fifty male subjects were enrolled in this study following these criteria, excluding those who refused to enroll in the study due to lack of enough time to attend therapy sessions and those who refused to sign the informed consent.

Then they were randomized into two groups, based on simple random sampling using an online randomizer (randomizer.org):

– **Group (A):** patients had 10 sessions of TPTNS on alternating days over a period of 3 weeks.
– **Group (B):** patients had 10 sessions of sham transcutaneous electrical nerve stimulation (TENS) on alternating days over a period of 3 weeks.

### The device parameters were set as follows

For the TPTNS group (group A), bilateral posterior tibial nerve stimulation was done using cup electrodes transmitting continuous current from Beurer® Em49 Digital TENS/EMS unit (Beurer GmbH, Ulm, Germany), the electrodes were placed along the course of posterior tibial nerve just behind the medial malleoli for 30 min with pulse width of 250 μs. The Em49 unit was used in high frequency set at 20 hz with the lowest amplitude needed to produce visual tetanic flexion of the big toe. This setting is enough to elicit retrograde stimulation sufficient for neuro-modulation with minimal side effects and good tolerance, as demonstrated in previous studies as Uribe et al. in 2020 [15].

While the sham TENS group (group B) used the same device but on the other side (behind lateral malleoli) away from the course of posterior tibial nerve with a lower amplitude not enough to induce any stimulation. Both groups were blinded to the intervention using the same device and same number and duration of sessions.

### Outcome measures

Clinical improvement was monitored using 7-item questionnaire developed by Arafa & Shamloul in 2007 known as AIPE [25], which assesses libido, erectile function, ejaculation time, control, satisfaction for the patient and partner as well as anxiety or depression. It classifies the severity of PE into five categories based on AIPE scores; No-PE (31–35), Mild (26–30), Mild to Moderate (20–25), Moderate (14–19), and Severe PE (7–13). The scale has been used and validated for use in the Middle East [26] and in Turkish population [27] with reliable outcomes.

The second outcome parameter was the IELT that was calculated using the digital hand watch. The patients were instructed to count the time between intromission and ejaculation, and to repeat this procedure in at least one coital occasion per week over 3 weeks of receiving sessions and to continue for 2 months after end of sessions.

Both outcome parameters AIPE & IELT along with IIEF-5 were registered at three time points, first was before starting treatment sessions (as the baseline assessment), second was after 3 weeks by the end of therapy (EOT), and the last was 2 months after the EOT for follow-up of cases who showed clinical improvement by EOT. Any adverse effect was noted during the course of the study.

#### The sample size estimation

We are planning a study of a continuous quantitative response variable (IELT and AIPE) in PE cases before and after treatment with TPTNS. The main outcome parameter is the difference in each paired parameter level in before vs after therapy, to be statistically compared between groups by Wilcoxon rank-sum test. Since this is the first RCT to be done on these variables in PE and we are only seeking detection of a clinically meaningful effect, a large effect size was presumed as per Cohen’s 1988 criteria, with effect size d = 0.85. A statistical power analysis was done for the estimation of the sample size; with a two-tailed alpha error probability set at 0.05, the predicted sample size which is needed for the effect size mentioned before (d = 0.85) is nearly *N* = 50 patients to be able to reject the null hypothesis that this difference is zero with probability (power) 0.8. With an allocation ratio of 1:1, each therapeutic arm will include 25 PE patients. To calculate the sample size G*Power 3.1.9.2 was used.

#### Statistical analysis

Data were coded and entered using the statistical package R studio version 1.0.143. Data were summarized using mean ± standard deviation in quantitative data and using frequency (count) and relative frequency (percentage) for categorical data. Median and interquartile range (IQR) were added to show the data which were not normal. Longitudinal comparisons between variables over time were done using paired non-parametrical Wilcoxon test. Mann–Whitney test was used to compare between quantitative variables in the two studied groups. Chi-square (χ2) test was performed to compare categorical data. Exact test was performed instead when the predicted frequency is less than 5. All analyses were done per protocol analysis except for improvement at EOT per AIPE, it was based on intention to treat analysis.

## Results

**Figure 1.**
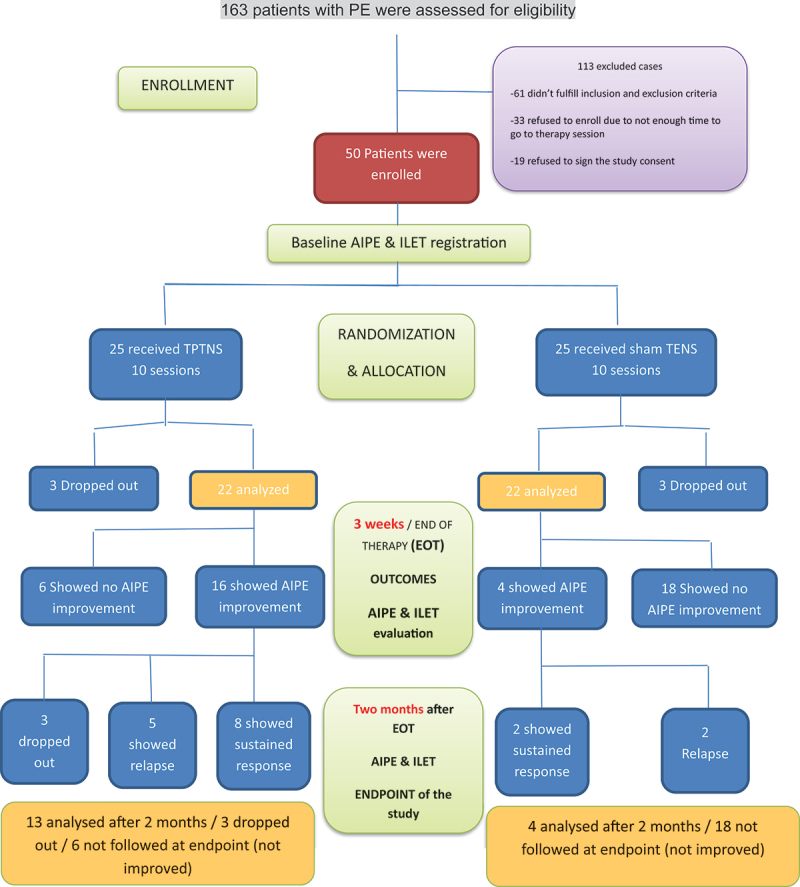
Patients’ flow diagram according to CONSORT guidelines for reporting randomized controlled trials.

Fifty patients fulfilled the criteria and were randomized to receive either TPTNS or sham TENS. Three patients dropped out during sessions schedule before EOT from each group. Figure 1 contains patients’ flow diagram according to the CONSORT guidelines for reporting randomized controlled trials.

As shown in Table 1, the baseline characteristics for the whole participants.

**Table 1.** Baseline characteristics for the whole participants and for each group.

	Intervention	nu	Median	IQR	Mean	Std. Deviation	P value (95% CI)
Age	TPTNS	25	31	7	31.32	6.19	0.173
	TENS	25	32	8	33.16	5.218	
	Total	50	31	6.75	32.24	5.74	
IELT	TPTNS	25	23	31	34.84	26.255	0.861
	TENS	25	27	26	31.04	19.097	
	Total	50	26.5	29.5	32.94	22.80	
IIEF	TPTNS	25	25	5	23.48	2.257	0.844
	TENS	25	25	5	23.2	2.449	
	Total	50	25	5	23.340	2.335	
AIPE	TPTNS	25	19	6	18.76	3.032	0.001
	TENS	25	15	2	16.04	2.245	
	Total	50	17	4.75	17.40	2.97	

For the entire population, the median (IQR) age was 31 (6.75), the baseline median (IQR) IIEF-5 score was 25 (5), the baseline median (IQR) AIPE score was 17 (4.75), and the baseline median (IQR) ILET in seconds was 26.50 s (29.50). The baseline characteristics were similar across both groups regarding the age (*p* = 0.173), baseline IIEF score (*p* = 0.844), and baseline ILET (*p* = 0.861) but not for the baseline AIPE (*p* = 0.001). However, both groups were within the moderate classification of PE according to Arafa & Shamloul in 2007, where the baseline median (IQR) AIPE score in TPTNS group was 19 (6) and the baseline median (IQR) AIPE score in sham TENS group was 15 (2).

At the EOT, treatment success was defined as AIPE score improvement and decrease in PE severity. The percentage of patients who achieved treatment success in the TPTNS group was significantly greater than in the TENS group; 64% (16/25 patients) versus 16% (4/25 patients) with *p* value = 0.001. This outcome was based on intention-to-treat analysis in which dropped out cases were considered as treatment failure, while according to per-protocol analysis, the percentage of improvement in TPTNS group was 72.72% (16/22 patients) versus 18.18% (4/22 patients) in TENS group (*p* < 0.001). Dropped out cases represented 12% (6/50) of the study population and 3/25 per each group.

**Figure 2.**
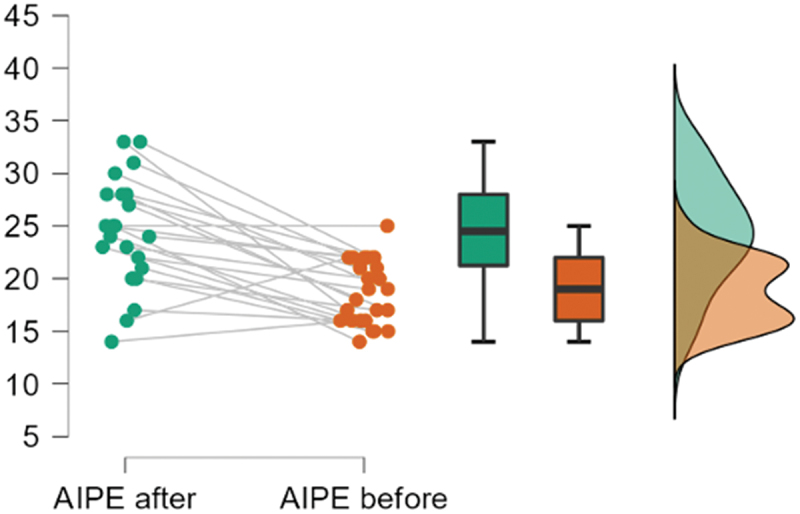
Raincloud plot of AIPE score in TPTNS group at baseline and at EOT.

The mean (SD) change from baseline in the AIPE score was 5.773 (4.105) in TPTNS group vs 1.273 (1.420) in the sham group while the median (IQR) was 6.000 (6.500) vs 1.000 (2.000), respectively, with a statistically significant difference between both groups (*p* < 0.001). This change manifested in improvement of PE category. Figure 2 presents the changes in the AIPE score in the TPTNS group.

**Figure 3.**
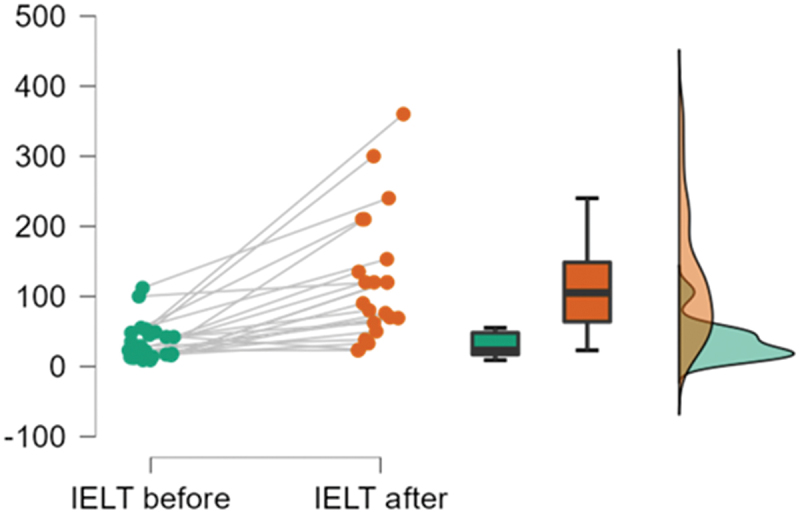
Raincloud plot of IELT in TPTNS group at baseline and at EOT.

The mean (SD) IELT fold change from baseline was 5.392 (5.507) in the TPTNS group vs 2.017 (1.316) in the TENS group while the median (IQR) was 3.095 (5.492) vs 1.230 (1.660), respectively, with a statistically significant difference between both groups (*p* = 0.01). Figure 3 presents IELT score changes in the TPTNS group. Regarding exceeding the IELT threshold, 11 patients (50%) in TPTNS group got ILET more than 2 min versus three cases (13.4%) in sham group with significant difference (*p* = 0.01).

The mean (SD) IIEF-5 score at EOT was 24.455 (1.101) in TPTNS and 24.045 (1.430) in the TENS group with no statistically significant difference between both groups (*p* = 0.389). Table 2 summarizes the results of our study.

**Table 2.** Comparison between both groups at EOT regarding AIPE score, IELT, and IIEF.

	TPTNS group	Sham TENS group	*p* value (95% CI)
Mean AIPE at EOT Median (IQR)	24.409 ± 5.179 24.500 (6.750)	17.364 ± 2.381 17.000 (2.750)	*<0.001*
Mean change from baseline Median (IQR)	*5.773 ± 4.105* *6.000 (6.500)*	*1.273 ± 1.420* *1.000 (2.000)*	*<0.001*
Mean % change from baseline Median (IQR)	*31.7021 ± 24.743* 29.167 (35.698)	*8.420 ± 9.832* 6.458 (13.125)	*<0.001*
Mean IELT at EOT (seconds) Median (IQR)	122.864 ± 90.620 105.000 (84.750)	49.909 ± 30.463 44.000 (18.250)	*<0.001*
Mean change from baseline Median (IQR)	*88.136 ± 88.370* *61.000 (83.750)*	*18.500 ± 24.850* *5.500 (27.500)*	*0.001*
Mean IELT fold change from baseline Median (IQR)	*5.392 ± 5.507* 3.095 (5.492)	*2.017 ± 1.316* 1.230 (1.660)	*0.010*
Mean IIEF at EOT Median (IQR)	24.455 ± 1.101 25 (0)	24.045 ± 1.430 25 (3)	*0.389*

*EOT; end of therapy*.

*p value is considered significant if < 0.05, Mann–Whitney U test.

In the 2-month follow-up of patients who achieved improvement at EOT (16 patients in the TPTNS group versus 4 patients in the TENS group), 50% of each group reported sustained improvement, 31.25% (5/16) of the TPTNS group failed to maintain improvement compared to 50% (2/4) of the TENS group (*p* = 0.585). 18.75% (3/16) of the TPTNS group dropped out from the follow-up.

Regarding adverse events, 2 out of 22 patients in TPTNS group (9.1%) reported mild side effects, one reported mild itching in the first session that disappeared later and the second reported hyperesthesia which occurred after five sessions and also disappeared in subsequent session without any intervention. While no adverse events were reported in the TENS group with no significant difference between both groups (*p* = 0.351).

## Discussion

Among all diverse treatment modalities for PE whether pharmacological, behavioral, or interventional, the peripheral nerve stimulation therapy has emerged recently as a new potential tool that can play an important role in management of this condition.

TPTNS is a non-invasive peripheral stimulation therapy that uses low-voltage electrical current to provide neuro-modulation and has long been established in pain management, rehabilitation of osteoarthritis, nerve injuries, and neuropathies [28].

TPTNS has been studied for the treatment of PE and it showed promising results in phase II clinical trial that was conducted in 2017–2018 by Uribe et al. The study revealed that TPTNS therapy was effective in delaying ejaculation in men with lifelong PE. The trial included 11 men who received three TPTNS therapies per week for 12 weeks. The results showed that the mean IELT increased from 1.7 min at baseline to 5.3 min at EOT showing the potential of TPTNS as a new treatment option for PE [15].

However, it had some limitations as small sample size, lack of control group, and using only the increase in IELT as definition of treatment success. To overcome these limitations, we used blinded controlled study design and defined treatment success by improvement of the multi-dimensional validated AIPE score and decrease of PE severity.

The results of our study revealed a higher statistically significant improvement in both AIPE score and IELT change from baseline among TPTNS group compared to sham TENS group (control arm) (*p* < 0.001).

Our results showed 64% improvement which were quite similar to the first trial of Uribe et al. that showed 54.5% of the participants improved [15]. However, the clinical improvement in their study was defined as increase in the baseline IELT by 3 folds while in our study treatment success was defined by AIPE score improvement and decrease in PE severity. Another important difference was in the treatment protocol, our participants received only 10 sessions over 3 weeks while in first trial done by Uribe et al. sessions continued for 3 months.

Fifty percent of the patients that underwent TPTNS therapy in our study were able to exceed the IELT cutoff value of 2 min at EOT with median change around 61 s and median IELT fold-increase from baseline around **3.095**. We depended on median values to robustly reflect the central tendencies of the skewed distribution. This was also comparable to the first trial in which geometric mean IELT increased by **4.82** fold at EOT [15].

In Uribe et al. study, the improvement was sustained in all cases after 3 months of finishing the therapy while in our study, the improvement was maintained in only 50% of patients after 2 months of end of treatment which might be due to the difference in treatment duration which allows cortical conditioning and plastic reorganization of cortical network.

Another study was conducted in 2020 by Aydos et al. to assess the role of TPTNS treatment in comparison to sham therapy in a group of 60 patients suffering from PE [29]. They reported comparable improvement in AIPE score, which improved in the TPTNS group from mean score 16.55 ± 4.74 to 21.4 ± 4.98 (*p* < 0.001). While the sham group showed less improvement from mean score 18.2 ± 5.2 to 20 ± 4.65 (*p* = 0.001). Differences from our study include that sessions were applied for a period of 12 weeks, but frequency was only once weekly and the sham device was applied without giving signals. In terms of the percentage change in IELT scores from pre-to-post procedure, TPTNS group was not statistically significant different from patients treated with sham (0.38 ± 0.47 vs 0.23 ± 0.67, *p* = 0.415) [29]. This is in contrast to our results in which percentage of IELT change were significantly increased after TPTNS therapy (*p* value = 0.001) and it is a matter of inquiry if the difference in frequency of sessions could explain this. Another limitations of the previous study was depending on the arithmetic mean for comparison and the lack of blindness as well as randomization.

Comparing TPTNS therapy to pharmacological modalities, it was found that the median fold increase in IELT post-TPTNS is comparable to numerous clinical trials that demonstrated that on-demand dapoxetine treatment is associated with a three-to-four fold increase in baseline IELT [30].

Furthermore, the improvement was maintained in 50% of the patients after 2 months of completing therapy, in contrast to the immediate loss of improvement typically observed after suspending pharmacological treatment [30].

Another advantage for TPTNS compared to pharmacological treatment is that all sessions of treatment were well tolerated by the patients and no side effects affected adherence to the therapy. Minor side effects were reported by only two patients (9.1%) in the form of itching and hyperesthesia, both were self-limited and did not affect their continuity on sessions. That goes in line with what was reported by Uribe et al., in which only two minor adverse events occurred in form of constipation and heat in perineum and also did not affect patient adherence to therapy [15]. In contrary to pharmacological treatments where patients experience numerous side effects such as dizziness, diarrhea, headache, nausea, dry ejaculation, decreased desire, or erection problems, which interfere with their compliance to treatment [31].

Also, in many cases, sexual acts are unplanned and treatments that need anticipated planning of sexual activity of 30 min or longer could compromise the natural spontaneity of sex and, therefore, might affect acceptance of the patient [32]. On the other side, TPTNS does not require on demand treatment improving the spontaneity of sexual life, patient compliance to therapy, and satisfaction.

On the other hand, 6/50 (12%) patients in our study dropped out due to inconvenience of three sessions per week in outpatient clinic which is in concordance with Uribe et al. study that reported a dropout rate of 8.3%. This finding favors the home care therapy to improve patient compliance and prolong the therapy duration.

### Limitations

Using arithmetic mean not geometric is considered one of the study limitations. However, we used mainly the median in all comparisons regarding IELT or AIPE score to reflect the central tendencies of the skewed distribution. There was a statistical difference in the baseline AIPE score between the two groups, however, this difference was unavoidable due to randomization process, also there was no difference in baseline IELT or PE category according to AIPE score categories. Concealment of allocation was not done. Follow-up period might be not long enough to assess the long-term effect of TPTNS.

## Conclusion

The current study provided us with additional clinical evidence for a new treatment concept for PE by TPTNS as it clearly showed that TPTNS is considered safe noninvasive procedure which was well tolerated by the patients and has good safety profile with significant positive effect on both IELT and AIPE scores.

## Recommendations

TPTNS has promising potential to be one of the reliable lines of treatment for PE. We need prospective RCTs comparing TPTNS to other treatment modalities with larger numbers of patients and for a prolonged follow-up periods for further evaluation of its efficacy and long-term effect.

## Abbreviations

AIPE: The Arabic index of premature ejaculation
EOT: End of therapy
IELT: Intra-vaginal ejaculatory latency time
ILEF-5: International Index of Erectile function-5
ISSM: The International Society for Sexual Medicine
IQR: Interquartile range
PDE-5i: Phosphodiesterase type 5 inhibitors
PE: Premature ejaculation
RCT: Randomized controlled trial
SSRIs: Selective serotonin reuptake inhibitors
TENS: Transcutaneous electrical nerve stimulation
TPTNS: Transcutaneous posterior tibial nerve stimulation
